# Supplementary material for: The causal role of circulating inflammatory markers in osteoporosis: a bidirectional Mendelian randomized study
Source: Front Immunol. 2024 Jul 18;15:1412298. doi: 10.3389/fimmu.2024.1412298 (PMC11291241; doi:10.3389/fimmu.2024.1412298)
Supplement: Supplementary file 1 [file DataSheet_1.zip › supplementary material/CIM-OSTEOPOROSIS /GCST90274802.csv_leave_one_out.pdf]

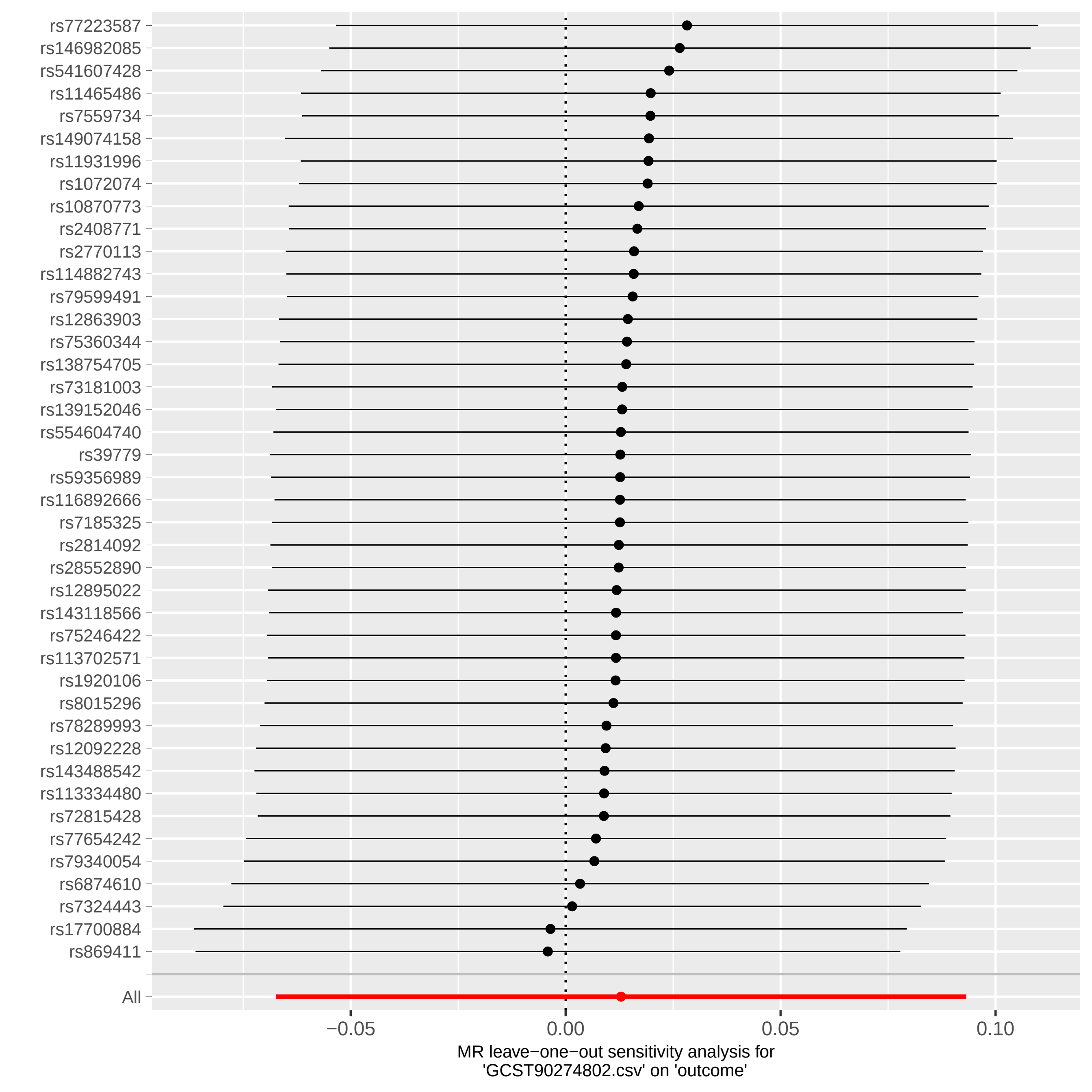
rs77223587
rs146982085
rs541607428
rs11465486
rs7559734
rs149074158
rs11931996
rs1072074
rs10870773
rs2408771
rs2770113
rs114882743
rs79599491
rs12863903
rs75360344
rs138754705
rs73181003
rs139152046
rs554604740
rs39779
rs59356989
rs116892666
rs7185325
rs2814092
rs28552890
rs12895022
rs143118566
rs75246422
rs113702571
rs1920106
rs8015296
rs78289993
rs12092228
rs143488542
rs113334480
rs72815428
rs77654242
rs79340054
rs6874610
rs7324443
rs17700884
rs869411
All
−0.05
0.00
0.05
0.10
MR leave−one−out sensitivity analysis for
'GCST90274802.csv' on 'outcome'
